# Genetic Analysis with Random Amplified Polymorphic DNA of the Multiple Enterocin-Producing* Enterococcus lactis* 4CP3 Strain and Its Efficient Role in the Growth of* Listeria monocytogenes* in Raw Beef Meat

**DOI:** 10.1155/2018/5827986

**Published:** 2018-06-10

**Authors:** Olfa Ben Braïek, Slim Smaoui, Karim Ennouri, Khaled Hani, Taoufik Ghrairi

**Affiliations:** ^1^Laboratory of Microorganisms and Active Biomolecules (LMBA), Faculty of Sciences of Tunis, University of Tunis El-Manar, Tunisia; ^2^Research Laboratory of Environmental Science and Technology (RLEST), ISSTE, Technopole de Borj Cedria, Tunisia; ^3^Laboratory of Microorganisms and Biomolecules of the Centre of Biotechnology of Sfax, Tunisia; ^4^UR012-ES03, Department of Biochemistry, Faculty of Medicine Ibn El Jazzar of Sousse, Tunisia

## Abstract

In this manuscript, a multiple enterocin-producing* Enterococcus lactis* strain named 4CP3 was used to control the proliferation of* Listeria monocytogenes* in refrigerated raw beef meat model. Also, the intraspecific genetic differentiation of 4CP3 strain was assessed by Random Amplified Polymorphic DNA Polymerase Chain Reaction (RAPD-PCR) analysis.* E. lactis* 4CP3 strain was found to produce the enterocins A, B, and P. It displayed activity against* L. monocytogenes* EGDe 107776 by agar-well diffusion method. The application of* E. lactis* 4CP3 culture at 10^7^ CFU/g in raw beef meat was evaluated using both ANOVA and ANCOVA linear models in order to examine its effect on the growth of the pathogen* L. monocytogenes* during refrigerated storage. Hence, a very interesting result in decreasing (*P*<0.05) and suppressing the growth of* L. monocytogenes* in refrigerated raw beef meat was shown during 28 days of storage. In conclusion,* E. lactis* 4CP3 strain might be useful for prevention of the proliferation and survival of* L. monocytogenes* in raw meat during refrigerated storage.

## 1. Introduction

Contamination and growth of* Listeria monocytogenes* in raw beef meats during refrigerated storage have been intensively reported [1–4]. Effectively,* L. monocytogenes* is known to be a major concern for the meat processing industry causing listeriosis in humans [4, 5]. This fact constitutes a significant public health issue [6]. Indeed, this virulent foodborne pathogen is psychrophile which is able to grow at temperatures as low as 0°C, adapted to several food systems and the contaminated foods do not present unusual odour, texture, or taste which evade control in human foodstuffs and increase its danger in products [7]. In this context, many researches were performed in order to develop natural agents other than antibiotics and chemically synthesised additives to ensure the safety and maintain the security of foods as public health issues [8]. Among these natural agents, lactic acid bacteria (LAB) have received great attention in terms of food safety and are mainly used in foods for different technological effects because of their potent Generally Recognised as Safe (GRAS) status [9]. In fact, LAB are implicated in the biopreservation and prolongation of the shelf-life of diverse food products owing to their production of antimicrobial substances [10, 11].

Bacteriocins are among the most studied antimicrobial substances produced by LAB [12]. These antimicrobial peptides (bacteriocins) may be added as biopreservatives to improve the microbial stability and safety of chill-stored fresh meat [13, 14]. Among the studied bacteriocins in meat and meat products we can cite the nisin. Produced by* Lactococcus lactis*, nisin was used successfully as food preservative in more than 50 countries [4]. This purified bacteriocin, nisin, showed bactericidal effect against* Listeria monocytogenes* in fresh meat and its application at 500 IU/ml engenders a significant reduction in the* L. monocytogenes* in meat [4]. On the other hand, direct use of bacteriocin-producing cells is one of the most practical strategies that seem to be more feasible from an economic point of view and lesser legal restrictions compared to the direct addition of purified bacteriocins. This can benefit the food industry in terms of microbiological quality and safety as well as cost since it reduces food losses caused by microbiological spoilage. Enterococci, isolated from diverse food sources, are among the most evaluated LAB as protective cultures in different foods due to their produced bacteriocins that are able to inhibit several key foodborne pathogens such as* L. monocytogenes *[15, 16]. Effectively, there are many strains of* Enterococcus* spp. that have been applied to the control of* L. monocytogenes* in different food systems [17–19]. Nowadays, advanced technologies have been developed for starters and protective cultures to enhance their efficacy and applicability in food products such as bioactive packaging and encapsulation [20]. Even though enterococci have been found naturally in different types of foods, their use in food products is controversial because they are considered as opportunistic pathogens implicated in several nosocomial infections and constitute a source of multiple antibiotic resistances [16].

The objectives of this work were to characterise genetically the multiple enterocin-producing* Enterococcus lactis* 4CP3 strain using RAPD-PCR analysis and evaluate its effect on the growth of* L. monocytogenes* in refrigerated raw beef meat.

## 2. Material and Methods

### 2.1. Strains and Growth Conditions

*E. lactis* 4CP3 strain was isolated from a raw shrimp (*Palaemon serratus*). The kinetic of bacteriocin production by 4CP3 strain was evaluated in MRS (de Man, Rogosa and Sharpe, Biokar Diagnostics, Beauvais, France) broth under aerobic conditions at 30°C [21]. This isolate was a multiple enterocin-producing strain able to produce the enterocins A, B, and P [21]. Also, it has been shown to display bactericidal mode of action against the pathogenic Gram-positive strain of* L. monocytogenes* EGDe 107776. It was grown overnight in MRS broth at 30°C.

*E. faecium* VC185 strain was isolated from Italian cheese [22]. This isolate is a non-bacteriocin-producing strain and is used in this study as the control strain in the meat challenge experimentation. It was also grown in MRS broth.

*L. monocytogenes* EGDe 107776 strain was used as the indicator strain for antimicrobial activity tests and the target microorganism in the microbiological challenge test. It was grown in BHI (Brain Heart Infusion, Biokar Diagnostics, Beauvais, France) broth and cultured on ALOA (Agar* Listeria* Ottaviani and Agosti, BIO-RAD, Marnes-la-Coquette, France) medium for enumeration [23].

*E. faecium* MMT21 strain was isolated from Tunisian rigouta cheese [24]. This isolate is used as the target strain in the direct detection of antimicrobial activity by overlaying with MRS soft agar in order to examine the capacity of* E. lactis* 4CP3 to produce bacteriocins in beef samples during the challenge test.

### 2.2. Random Amplified Polymorphic DNA-PCR (RAPD-PCR) Analysis

Genomic DNA used for RAPD-PCR amplification was extracted from overnight culture of* E. lactis* 4CP3 in M17 broth at 30°C according to Cremonesi et al., 2006 [25]. RAPD-PCR amplification was realised using the universal primers M13 and D8635 as described by Andrighetto et al., 2001 [26]. Amplification products were separated by electrophoresis on agarose gel (1.5%) in 1 × TAE buffer at 100 mV for 99 min. The gels were stained in ethidium bromide and photographed on a UV transilluminator. Photo-positives were scanned into a computer and were analysed using the BioNumeric 5.0 software package (Applied Maths NV, Sint-Martens-Latem, Belgium). Grouping of the RAPD-PCR patterns was performed using the Unweighted Pair Group Method with Arithmetic Averages (UPGMA) cluster analysis. The reproducibility value of the RAPD-PCR assay, calculated from two repetitions of independent amplification of type strains, was higher than 90%. The RAPD-PCR profiles obtained with both primers (M13 and D8635) were analysed together to obtain a single dendrogram.

### 2.3. Antimicrobial Activity against* L. monocytogenes*

Overnight culture of* E. lactis* 4CP3 strain incubated at 30°C in MRS broth was centrifuged at 10,000 ×* g* for 10 min to obtain a cell free supernatant which was neutralised at pH 6 with NaOH (1 M) in order to eliminate the inhibitory effect of organic acids, and sterilised by filtration (0.22 *µ*m, Millipore, Bedford, MA) [21]. The antimicrobial activity of the cell free supernatant of* E. lactis* 4CP3 against* L. monocytogenes* EGDe 107776 was assayed by the agar-well diffusion method according to Ben Braïek et al., 2017 [27]. The BHI agar plate was incubated at 37°C for 24 h and the diameter of the inhibition zone was measured in millimetres (mm).

### 2.4. Influence of* E. lactis* 4CP3 Strain addition on the Growth of* L. monocytogenes* EGDe 107776 in Raw Beef Meat

#### 2.4.1. Preparation and Inoculation of Raw Beef Samples

Raw beef meat was bought from a local supermarket in the region of Sousse (Tunisia) and transported to the laboratory under refrigerated conditions to be processed immediately. The prepared beef meat was aseptically cut into five equal portions of 200 g each (BF1-BF5). In order to reduce to the lowest possible levels the number of intrinsic microorganisms attached to the surface of beef meat portions, each piece was immersed in boiling sterile water for 5 min [28]. The cooked surface of the meat samples was eliminated with sterile knives under aseptic conditions [28]. These meat portions were further cut into small pieces of about 2 × 2 × 0.5 cm. Prior to beef meat contamination with* L. monocytogenes* and inoculation with LAB strains, beef portions were examined for any contamination by mesophilic and psychrotrophic flora. Total mesophilic bacteria were determined on plate count agar (PCA; Difco Laboratories, Detroit, MI, USA), incubated at 30°C for 48 h. Psychrotrophic counts were determined as described above for mesophilic bacteria except that the incubation was at 4°C for 7 days [29].

*E. lactis* 4CP3 strain was grown in MRS broth at 30°C for 24 h to reach the maximum of its bacteriocin production (1400 AU/ml) [21].* L. monocytogenes* EGDe 107776 was subcultured in BHI broth firstly at 37°C for 18 h to reach the early stationary phase with cells at the same physiological state, then at 10°C (temperature of the meat storage) for 3 days as adaptation step to the storage conditions. The* in situ* influence of the application of* E. lactis* 4CP3 strain on the survival of* L. monocytogenes* EGDe 107776 in raw beef meat was assessed according to the slightly modified method of Dortu et al., 2008 [6]. Briefly, the portions BF2 and BF3 were firstly surface inoculated at 10^7^ CFU/g of meat with* E. lactis* 4CP3 and* E. faecium* VC185 strains, respectively. After absorption of the LAB inocula at room temperature, the BF1, BF2, and BF3 meat portions were surface contaminated with 10^5^ CFU of* L. monocytogenes* EGDe 107776/g of meat. A sterile spreader was used to distribute homogeneously the inocula. The portion BF1 served as control (artificially contaminated only with* L. monocytogenes* EGDe 107776). The portion BF4 and BF5 were not contaminated with* L. monocytogenes* EGDe 107776 but were inoculated only with 4CP3 and VC185 strains, respectively, at 10^7^ CFU/g of meat.

#### 2.4.2. Storage and Enumerations

The raw beef meat portions were placed separately in sterile plastic bags and stored for 28 days at 10°C. The choice of this storage temperature relies firstly on the growth conditions of enterococcal strains used in this study that are not able to grow at temperatures lower than 10°C [30]. Secondly, meat storage at 10°C aimed to mimic the worst-case scenario for cold storage according to Kennedy et al., 2005 [31].

*L. monocytogenes* EGDe 107776 plate counts were determined on ALOA agar plates according to NF EN ISO 11290-2: 2005 [23]. The LAB counts were determined on MRS agar plates after incubation at 30°C for 24 h. Microbial enumerations were expressed as log_10_ CFU/g of beef meat. Plates containing 25-250 colonies were selected and counted, and the average number of CFU/g was calculated. These cell counts were performed every 6 h during 48 h, every day up to day 7 and every 7 days until day 28.

To detect enterocin production by* E. lactis* 4CP3 in raw beef meat, homogenates from the portions BF2 and BF4 stored at days 0, 7, 14, 21, and 28 were plated on MRS agar. After aerobic incubation at 30°C for 24 h, the plates were further overlaid with the indicator strain* E. faecium* MMT21 in soft agar and incubated overnight at 30°C. Bacteriocin production was indicated by clear inhibition zones around the colonies.

### 2.5. Statistical Analysis

Measurements were carried out in triplicates and repeated three times. A one-way analysis of variance (ANOVA) was applied for each parameter by using SPSS 19 statistical package (SPSS Ltd., Woking, UK). Means and standard errors were calculated and a probability level of* P*<0.05 was used in testing the statistical significance of all experimental data. Tukey's post hoc test was used to determine significance of mean values for multiple comparison at* P*<0.05. On the other hand, we used linear mixed models assuming the error to compare the CFU values among treatments with different days. Mixed models were fitted using SPSS 19 and followed by post hoc contrasts through the origin. The interpretation of the statistical output of a mixed model requires an understanding of how to explain the relationships among the fixed and random effects in terms of the hierarchy levels.

## 3. Results and Discussion

### 3.1. RAPD-PCR Analysis

**Figure 1:**
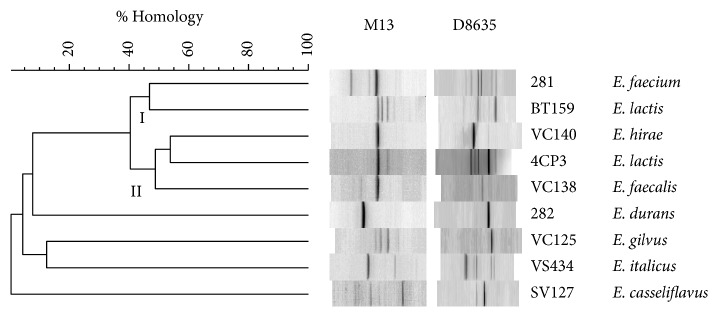
Unweighted pair group method using arithmetic averages (UPGMA) based dendrogram derived from the combined RAPD-PCR profiles generated with primers M13 and D8635 of* E. lactis* 4CP3 strain, type strains, and other enterococcal strains. The type strains used in this analysis were* E. lactis* DSM 23655^T^ (BT159),* E. faecium* DSM 20477^T^ (281), and* E. durans* DSM 20633^T^ (282) from the Deutsche Sammlung von Mikroorganismen und Zellkulturen, Braunschweig (Germany). The other enterococcal strains were* E. gilvus* (VC125),* E. italicus* (VS434),* E. hirae* (VC140),* E. faecalis* (VC138), and* E. casseliflavus* (SV127) from the bacterial collection of ISPA-CNR (Milan, Italy).

*E. lactis* 4CP3 strain was previously identified by different genetic methods: 16S rRNA gene sequencing,* rpoA* and* pheS* gene sequencing, and 16S-23S rRNA intergenic spacer analysis (RSA) [21]. Indeed, RSA analysis demonstrated that* E. lactis* 4CP3 strain displayed the same 16S-23S profile as the type strain* E. lactis* DSM 23655^T^ (BT159), while in this study, they presented different RAPD-PCR patterns as shown in Figure 1. Accordingly, two clusters (I and II) could be detected at a similarity level of 45% arbitrarily chosen for defining species. Interestingly,* E. lactis* 4CP3 and the type strain* E. lactis* BT159 were found to belong to different clusters even though they belong to the same species (Figure 1). This genetic differentiation between* E. lactis* 4CP3 and BT159 strains as illustrated by their clustering in the dendrogram could be related to their different isolation sources. Effectively, our* E. lactis* 4CP3 strain was isolated from a fresh shrimp sample of* Palaemon serratus* [21], while,* E. lactis* BT159 strain was isolated from an Italian cheese sample [32]. Therefore, RAPD-PCR analysis constitutes a rapid molecular method that could detect genetic diversity at a strain level with accuracy.

### 3.2. Antilisterial Activity

*In vitro* antibacterial assay of* E. lactis* 4CP3 strain showed high antilisterial activity (*P*<0.05) against* L. monocytogenes* EGDe 107776 with a clear growth inhibition zone diameter of 12 mm on BHI agar (Table 1). This result corroborates previous finding described for* E. faecium* strains [17]. This antagonistic activity towards* L. monocytogenes* was due to the production of the enterocins (A, B, and P) as previously demonstrated by Ben Braïek et al., 2018 [21]. In fact, the enterocins A, B, and P are among the most characterised bacteriocins and are known to be active against* Listeria* spp. as reported by Vandera et al. 2017 [16] and Rehaiem et al. 2014 [33].

### 3.3. Influence of* E. lactis* 4CP3 Strain addition on the Growth of* L. monocytogenes* EGDe 107776 in Raw Beef Meat Using ANOVA

Meat is considered to be one of the most frequently contaminated foods with* L. monocytogenes* [3]. According to Rapid Alert System for Food and Feed in 2016 [34], 20% of* L. monocytogenes* notifications were due to the contamination of meats other than poultry. In this context, a challenge test to control the growth of* L. monocytogenes* in raw beef meat inoculated with an enterocin-producing* E. lactis* strain was carried out. Furthermore, it is important to mention that high levels of intrinsic nonpathogenic microorganisms may have an inhibitory effect on pathogens present in meat by outcompeting them [28]. For this reason, our meat samples were subjected to boiling treatment with sterile water as described above in order to reduce the number of factors that could be implicated in the listerial growth in beef food models and to ovoid interferences of colonies on plating agar.

It should be noted that the analysis of mesophilic and psychrotrophic bacteria from meats treated separately with* E. lactis* 4CP3 and* E. faecium* VC185 strains showed an inhibition of these bacteria (mesophilic and psychrotrophic) since only the LAB,* E. lactis* and* E. faecium*, were identified (data not shown). In fact, the microbial load of aerobic mesophilic plate count and psychrotrophic count was zero, demonstrating the effective process of the boiling sterile water immersion intervention to eliminate these bacteria (aerobic mesophilic and psychrotrophic) from meat portions.

**Figure 2:**
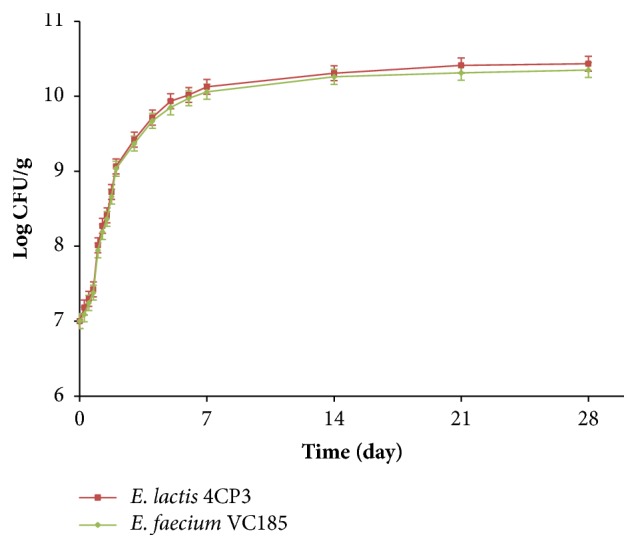
Growth of LAB strains in raw beef meat. Red square:* E. lactis* 4CP3 (enterocin-producing LAB strain) and green diamond:* E. faecium* VC185 (non-bacteriocin-producing LAB strain).

**Figure 3:**
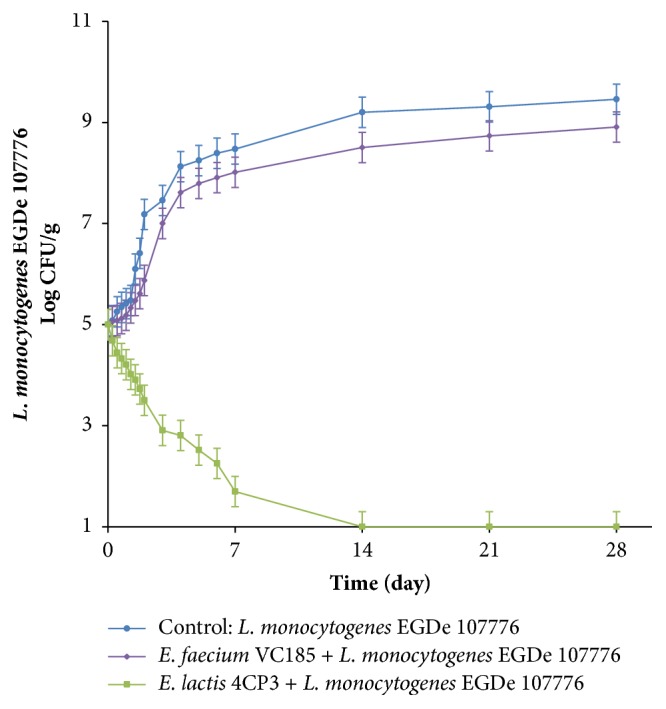
Influence of inhibitory LAB cultures on the growth of* L. monocytogenes* EGDe 107776 in raw beef meat during storage at 10°C. Blue circle: control (*L. monocytogenes* EGDe 107776 without enterocin-producing LAB strain), violet diamond:* E. faecium* VC185 (non-bacteriocin-producing LAB strain), and green square:* E. lactis* 4CP3 strain (enterocins A, B and P-producing strain).

As demonstrated by Figure 2, there were no significant differences (*P*>0.05) in the growth of* E. lactis* 4CP3 strain and* E. faecium* VC185 strain in raw beef meat showing comparable growth rates increasing by 3.43 logs and 3.35 logs, respectively, in 28 days of storage. The population of* L. monocytogenes* EGDe 107776 in portion BF1 (positive control: artificially contaminated with 10^5^ CFU/g of meat) underwent an increase from 10^5^ CFU/g to 2.87×10^9^ CFU/g after 28 days (Figure 3).

Statistical evaluation of the data relating to the growth behaviour of* L. monocytogenes* EGDe 107776 in raw beef meat inoculated with* E. lactis* 4CP3 strain showed significant reduction (*P*<0.05) of listerial population by 6.77 log units compared with the untreated control after 7 days of storage (Figure 3). Then, the growth of* L. monocytogenes* was completely inhibited from day 14 to the end of the experiment.

The application of the non-bacteriocin-producing* E. faecium* VC185 strain led to a very low reduction of* L. monocytogenes* populations. These counts were only 0.46 log units and 0.55 log units lower than the control counts after 7 and 28 days of storage, respectively. Moreover, no significant growth (*P*>0.05) of* L. monocytogenes* EGDe 107776 was observed in the portions BF4 and BF5 which were only inoculated with LAB strains at 10^7^ CFU/g and not contaminated with the listerial pathogen.

### 3.4. *In Situ* Detection of Enterocin Production in Raw Beef Meat

Overlay assays with MRS agar plates were realised in order to detect* in situ* production of enterocins by* E. lactis* 4CP3 strain in beef meat samples during the refrigerated storage period. After incubation, enterocin production was indicated by observation of obvious inhibition zones around the colonies grown on MRS agar medium. Generally, it was shown that the application of the multiple enterocin-producing* E. lactis* 4CP3 strain in raw beef meat led to a greater (*P*<0.05) inhibition of* L. monocytogenes* EGDe 107776 than that of the non-bacteriocin-producing* E. faecium *VC185 strain (Figure 3). Also, it was demonstrated in this study that this enterococcal culture strongly (*P*<0.05) inhibited the growth of* L. monocytogenes* in beef meat after the first 7 days of the challenge test and then suppressed dramatically the pathogen. This potent inhibitory behaviour of* E. lactis* 4CP3 towards* L. monocytogenes *could be explained by the enterocin production as confirmed above. In fact, enterocins A and P have strong antilisterial activity against* L. monocytogenes*; however, enterocin B displays synergistic activity with enterocin A [16, 33]. Thus, our present results corroborate these previous findings indicating that enterocins A and B may synergistically inhibit* L. monocytogenes* growth. Likewise, a synergistic interaction between the three produced enterocins (A, B, and P) by* E. lactis* 4CP3 could be proposed reflecting thus its effectiveness in raw beef meat preservation. Similar results reporting the biocontrol of* L. monocytogenes* in different meat products with bacteriocinogenic LAB were previously described by Dortu et al., 2008 [6], Pragalaki et al., 2013 [35], and Giello et al., 2018 [36]. Therefore, it is clear that application of bacteriocin-producing LAB in meats and meat products have been attracting considerable interest as alternative natural food preservatives to extend shelf-life and safety of meats these recent years [37, 38]. Effectively, direct application of bacteriocin-producing LAB is among the most advanced and practical approaches from economic and regulatory status point of views. Indeed, this bacterial use does not need many processing steps such as purification and has fewer legal restrictions and limits compared to the direct application of purified bacteriocins [39].

### 3.5. Influence of* E. lactis* 4CP3 Strain addition on the Growth of* L. monocytogenes* EGDe 107776 in Raw Beef Meat Using General Linear Model (ANCOVA)

Analysis of covariance (ANCOVA) is a general linear model which blends ANOVA and regression. ANCOVA evaluates whether the means of dependent variables (11 sampling days: 0, 1, 2, 3, 4, 5, 6, 7, 14, 21, and 28 days of storage at 10°C) which are equal across levels of categorical independent variables (five trials: Trial 1: BF1, Trial 2: BF2, Trial 3: BF3, Trial 4: BF4, and Trial 5: BF5) and inversely. In order to simplify the obtained results, for each meat product, firstly (i) we analysed parameters between 0 and 7 days and secondly (ii) all parameters were evaluated between 7 and 28 days.

#### 3.5.1. ANCOVA Parameter Analyses between 0 and 7 Days

As in ANCOVA, writing out the full regression model and then simplifying tells us that the intercept for day zero was 4.000 (4.194436–0.194436) and this was lower than log_10_ CFU at the seventh day group (t= -0.053). Similarly, we knew that the days 0, 1, 2, 3, 4, 5, and 6 had lower intercepts than the 7^th^ day. The trial coefficient of 1.160110 represented the average for each subsequent trial for the baseline on day 7. The interaction estimates tell the difference in slope for other day groups compared to the seventh day groups (Table 2(a)). We are particularly interested in the conclusion that we are 95% confident that the control sample had an effect on the CFU that was between 16.611213 points more and -17.731432 points less than treatment for beef meat (Table 2(a)).

Equally, ANCOVA indicated that there were no statistically significant differences (*P*>0.05) among the treatments between 0 and 7 days (Table 2(a)).

As shown in Table 2(b), writing out the full regression model then simplifying tells us that the intercept for trial 1 was 5.277044 (7.467589–2.190545). Similarly, we knew the trials 2, 3, 4, and 5. The day coefficient of 0.489768 represented the average for each subsequent trial for the baseline on the trial 5 (Table 2(b)). The interaction estimates tell the difference in slope for other trial groups compared to the fifth groups (Table 2(b)).

The treatments BF1 (control sample), BF3 (*E. faecium* VC185 strain at 10^7^ CFU/g of meat + 10^5^ CFU of* L. monocytogenes* EGDe 107776/g of meat), BF4 (only* E. lactis *4CP3 strain at 10^7^ CFU/g of meat), and BF5 (only* E. faecium* VC185 strain at 10^7^ CFU/g of meat) had no significant differences (*P*>0.05) between them. However, at the* P*<0.001 confidence level, the treatment of* E. lactis* 4CP3 strain at 10^7^ CFU/g of meat + 10^5^ CFU of* L. monocytogenes* EGDe 107776/g of meat (BF2) was statistically different and was more sensitive to dose than the other trials (Table 2(b)).

It is very important to realise that the parameter estimates given in the fixed effects were estimates of mean parameters. The covariance parameters are presented in Table 3. Equally, the intercepts' variances were estimated as 0.134603 and 6.125832 (Table 3). The null hypothesis for this parameter was a variance of zero, which would indicate that a random effect was not needed. The statistical test is called Wald Z statistic. On the other hand, the hypothesis (Wald Z = 0.000,* P* = 1.00) was accepted and the null hypothesis (Wald Z = 1.067,* P* = 0.286) was rejected. In fact, we conclude that we do need a random intercept (Table 3). This suggests that there are important unmeasured explanatory variables for each subject that raise or lower their performance in a way that appears random because we do not know the values of the missing explanatory variables.

#### 3.5.2. ANCOVA Parameter Analyses between 7 and 28 Days

For a period ranged between 7 and 28 days of storage, the ANCOVA intercept for day seven was 4.194435 (4.664446–0.470011) and this was lower than log_10_ CFU at the twenty-eighth day group (t= -0.081) (Table 4(a)). Similarly, the days 7, 14, and 21 had lower intercepts than the day 28. The trial coefficient of 1.121930 represented the average for each subsequent trial for the baseline on the day 28 (Table 4(a)). Furthermore, the treatment control sample (Trial 1) had an effect on the CFU (Table 4(a)).

As shown in Table 4(a), there were no significant differences (*P*>0.05) among the trials and days 7, 14, 21, and 28.

Indeed, the lower and upper bound of the confidence interval for the mean difference ranged from -25.174660 points to 25.251019 points (Table 4(a)). The full regression model then simplifying the intercept for the control sample was 8.344604 (10.015610–1.671006) (Table 4(b)). Similar results were shown for 2, 3, 4, and 5 trial groups. The day coefficient was 0.013178.

The effects of treatments, time, and their interaction on the inhibition of* L. monocytogenes* are shown in Table 4(b). no significant interaction (*P*>0.05) between treatments BF3 and BF4, and the time of storage in meat. However, interestingly, at the* P*<0.01 confidence level, BF2 and time of storage were found to have a highly significant effect regarding inhibition of* L. monocytogenes* EGDe 107776 in meat (Table 4(b)).

Moreover, the intercepts' variances were estimated as 0.029149 and 15.235632. Besides, the hypothesis (Wald Z = 0.000,* P* = 1.00) was accepted for beef meat samples (Table 5).

### 3.6. Practical Aspects

Enterococcal strains with a view to be used as protective or starter/adjunct cultures in biopreservation of foods, must usually be selected on the basis of the safety aspects which frequently are the absence of virulence and antibiotic resistance traits. Effectively,* E. lactis* 4CP3 strain was previously verified as nonhaemolytic, gelatinase negative, sensitive to vancomycin and other clinically relevant antibiotics and lacked known antibiotic resistance genes and several significant virulence factors [21]. Therefore, the presence of* E. lactis* 4CP3 in meat does not appear to represent a health risk.

## 4. Conclusion

To the best of our knowledge, this is the first report on the application of a multiple enterocin-producing* E. lactis* strain to control* L. monocytogenes* in artificially contaminated raw beef meat during refrigerated storage. Based on the obtained results,* E. lactis* 4CP3 strain might be useful as natural biopreservative against* L. monocytogenes* in meat products.

## Figures and Tables

**Table 1:** Inhibitory spectrum of *E. lactis* 4CP3, CR4, CL, 5CP2, C15, and C23 strains against *L. monocytogenes* EGDe 107776.

Test strain	Diameter of inhibition zones (mm)
*E. lactis* 4CP3	12.00±1.00^d^
*E. lactis* CR4	10.00±1.00^c^
*E. lactis* CL	5.00±0.00^b^
*E. lactis* 5CP2	5.00±0.00^b^
*E. lactis* C15	0.00±0.00^a^
*E. lactis* C23	0.00±0.00^a^
PC	18.00±2.00^e^
NC	0.00±0.00^a^

Results are reported as means ± standard error of three replicates.^a–e^: averages with different letters in the same column, for each diameter of inhibition zones, are significantly different (*P*<0.05). PC: positive control (Novobiocin 1 mg/ml) and NC: negative control (noninoculated MRS broth medium).

**Table:** (a) Raw beef meat estimates of trials fixed effects between 0 and 7 days.

Parameter	Estimate	Std. Error	Df	t	Sig.	95% Confidence Interval	
						Lower Bound	Upper Bound
Intercept	4.194436	2.595846	24	1.616	0.119 (ns)	-1.163127	9.551998
Day 0	-0.194436	3.671080	24	-0.053	0.958 (ns)	-7.771173	7.382302
Day 1	-0.699838	3.671080	24	-0.191	0.850 (ns)	-8.276576	6.876899
Day 2	-0.041189	3.671080	24	-0.011	0.991 (ns)	-7.617927	7.535548
Day 3	-0.064947	3.671080	24	-0.018	0.986 (ns)	-7.641685	7.511790
Day 4	0.392455	3.671080	24	0.107	0.916 (ns)	-7.184282	7.969193
Day 5	0.285756	3.671080	24	0.078	0.939 (ns)	-7.290981	7.862494
Day 6	0.235276	3.671080	24	0.064	0.949 (ns)	-7.341462	7.812013
Day 7	0^a^	0	.	.	.	.	.
Trial	1.160110	6.194913	94193.706	0.187	0.851 (ns)	-10.981849	13.302068
Day 0 × Trial	-0.560110	8.760930	94193.706	-0.064	0.949 (ns)	-17.731432	16.611213
Day 1 × Trial	-0.273876	8.760930	94193.706	-0.031	0.975 (ns)	-17.445198	16.897447
Day 2 × Trial	-0.233863	8.760930	94193.706	-0.027	0.979 (ns)	-17.405185	16.937460
Day 3 × Trial	-0.126228	8.760930	94193.706	-0.014	0.989 (ns)	-17.297550	17.045095
Day 4 × Trial	-0.160129	8.760930	94193.706	-0.018	0.985 (ns)	-17.331451	17.011194
Day 5 × Trial	-0.097362	8.760930	94193.706	-0.011	0.991 (ns)	-17.268685	17.073960
Day 6 × Trial	-0.067141	8.760930	94193.706	-0.008	0.994 (ns)	-17.238464	17.104181
Day 7 × Trial	0^a^	0	.	.	.	.	.

^a^ : this parameter is set to zero because it is redundant. Std. Error: standard error, df: the degrees of freedom, t: Student's t-statistic, and Sig.: the p-value (associated with the correlation). ns: *P*>0.05.

**Table:** (b) Raw beef meat estimates of days fixed effects.

Parameter	Estimate	Std. Error	df	t	Sig.	95% Confidence Interval	
						Lower Bound	Upper Bound
Intercept	7.467589	0.246726	26.643	30.267	0.000 (*∗∗∗*)	6.961032	7.974147
Trial 1	-2.190545	0.334916	23.708	-6.541	0.000 (*∗∗∗*)	-2.882229	-1.498862
Trial 2	-3.072563	0.334916	23.708	-9.174	0.000 (*∗∗∗*)	-3.764247	-2.380879
Trial 3	-2.595970	0.334916	23.708	-7.751	0.000 (*∗∗∗*)	-3.287653	-1.904286
Trial 4	0.028207	0.334916	23.708	0.084	0.934 (ns)	-0.663477	0.719891
Trial 5	0^a^	0	.	.	.	.	.
Day	0.489768	0.070900	21.369	6.908	0.000 (*∗∗∗*)	0.342479	0.637057
Trial 1 × Day	0.103902	0.080060	23.708	1.298	0.207 (ns)	-0.061443	0.269246
Trial 2 × Day	-0.836490	0.080060	23.708	-10.448	0.000 (*∗∗∗*)	-1.001834	-0.671146
Trial 3 × Day	0.080347	0.080060	23.708	1.004	0.326 (ns)	-0.084998	0.245691
Trial 4 × Day	0.005842	0.080060	23.708	0.073	0.942 (ns)	-0.159502	0.171186
Trial 5 × Day	0^a^	0	.	.	.	.	.

^a^ : this parameter is set to zero because it is redundant. Std. Error: standard error, df: the degrees of freedom, t: Student's t-statistic, and Sig.: the p-value (associated with the correlation). Trial 1: BF1 (control sample), Trial 2: BF2, Trial 3: BF3, Trial 4: BF4, and Trial 5: BF5. ns: *P*>0.05, *∗∗∗*: *P*<0.001.

**Table 3:** Estimates of covariance parameters in raw beef meat samples between 0 and 7 days.

Parameter		Estimate	Wald Z	Sig.
Residual		0.134603	3.443	0.001
Day [subject = id]	Variance	0.006163	1.067	0.286
Residual		6.125832	3.464	0.001
Trial [subject = id]	Variance	37.764364^a^	.	.

^a^ : this covariance parameter is redundant. The test statistic and confidence interval cannot be computed. Sig.: the p-value (associated with the correlation).

**Table:** (a) Raw beef meat estimates of trials fixed effects between 7 and 28 days.

Parameter	Estimate	Std. Error	Df	t	Sig.	95% Confidence Interval	
						Lower Bound	Upper Bound
Intercept	4.664446	4.093800	12	1.139	0.277 (ns)	-4.255177	13.584069
Day 7	-0.470011	5.789507	12	-0.081	0.937 (ns)	-13.084262	12.144241
Day 14	-0.239269	5.789507	12	-0.041	0.968 (ns)	-12.853521	12.374983
Day 21	-0.138680	5.789507	12	-0.024	0.981 (ns)	-12.752932	12.475572
Day 28	0^a^	0	.	.	.	.	.
Trial	1.121930	2.383010	0.000	0.471	1.000 (ns)	-16.706240	18.950100
Day 7 × Trial	0.038180	3.370085	0.000	0.011	1.000 (ns)	-25.174660	25.251019
Day 14 × Trial	0.021102	3.370085	0.000	0.006	1.000 (ns)	-25.191737	25.233942
Day 21 × Trial	0.020506	3.370085	0.000	0.006	1.000 (ns)	-25.192333	25.233345
Day 28 × Trial	0^a^	0	.	.	.	.	.

^a^ : this parameter is set to zero because it is redundant. Std. Error: standard error, df: the degrees of freedom, t: Student's t-statistic, and Sig.: the p-value (associated with the correlation).

ns: *P*>0.05.

**Table:** (b) Raw beef meat estimates of days fixed effects.

Parameter	Estimate	Std. Error	df	t	Sig.	95% Confidence Interval	
						Lower Bound	Upper Bound
Intercept	10.015610	0.209103	10	47.898	0.000 (*∗∗∗*)	9.549700	10.481519
Trial 1	-1.671006	0.295716	10	-5.651	0.000 (*∗∗∗*)	-2.329902	-1.012110
Trial 2	-8.316640	0.295716	10	-28.124	0.000 (*∗∗∗*)	-8.975536	-7.657744
Trial 3	-2.204440	0.295716	10	-7.455	0.000 (*∗∗∗*)	-2.863336	-1.545544
Trial 4	0.047959	0.295716	10	0.162	0.874 (ns)	-0.610937	0.706855
Trial 5	0^a^	0	.	.	.	.	.
Day	0.013178	0.010908	10	1.208	0.255 (ns)	-0.011126	0.037482
Trial 1 × Day	0.030534	0.015426	10	1.979	0.046 (*∗*)	-0.003837	0.064905
Trial 2 × Day	-0.043134	0.015426	10	-2.796	0.009 (*∗∗*)	-0.077504	-0.008763
Trial 3 × Day	0.028453	0.015426	10	1.845	0.095 (ns)	-0.005917	0.062824
Trial 4 × Day	0.001487	0.015426	10	0.096	0.925 (ns)	-0.032884	0.035857
Trial 5 × Day	0^a^	0	.	.	.	.	.

^a^ : this parameter is set to zero because it is redundant. Std. Error: standard error, df: the degrees of freedom, t: Student's t-statistic, and Sig.: the p-value (associated with the correlation). Trial 1: BF1 (control sample), Trial 2: BF2, Trial 3: BF3, Trial 4: BF4, and Trial 5: BF5. ns: *P*>0.05, *∗*: *P*<0.05, *∗∗*: *P*<0.01, and *∗∗∗*: *P*<0.001.

**Table 5:** Estimates of covariance parameters in raw beef meat samples between 7 and 28 days.

Parameter		Estimate	Wald Z	Sig.
Residual		0.029149	2.236	0.025
Day [subject = id]	Variance	0.000000^a^	.	.
Residual		15.235632	2.449	0.014
Trial [subject = id]	Variance	4.155172	0.000	1.000

^a^ : this covariance parameter is redundant. The test statistic and confidence interval cannot be computed. Sig.: the p-value (associated with the correlation).
